# Exposure Pathways to Antimicrobial Resistance at the Human-Animal Interface—A Qualitative Comparison of Swiss Expert and Consumer Opinions

**DOI:** 10.3389/fpubh.2020.00345

**Published:** 2020-07-30

**Authors:** Isabel Lechner, Claudia Freivogel, Katharina D. C. Stärk, Vivianne H. M. Visschers

**Affiliations:** ^1^SAFOSO AG, Bern, Switzerland; ^2^University of Applied Sciences and Arts Northwestern Switzerland, Olten, Switzerland

**Keywords:** AMR, transmission, risk, pets, animals, food safety, behavior

## Abstract

Antimicrobial resistance (AMR) is an emerging global health concern, affecting both the animal and the human population. Transmission pathways of AMR are therefore abundant and complex, and ways to prevent or reduce transmission to consumers must be identified. The overall goal of this study was to define the content of an intervention study aimed at reducing the transmission of AMR from animal sources to humans. To identify the most relevant pathways, Swiss experts and consumers were interviewed about their opinions on the risks of transmission of AMR. Opinions of experts and consumers were then qualitatively compared and the main gaps identified. The results revealed that Swiss consumers had several misconceptions regarding the sources and transmission of AMR, and that they in particular underestimated the importance of poultry meat and pets as a potential source of AMR. Furthermore, high uncertainty was noted in experts regarding the prevalence of AMR in pets and the potential of transmission to their owners. Consequently, awareness of AMR transmission pathways should be increased among consumers to overcome common misconceptions, which will help reduce the risk of transmission. Further research is needed to better understand the pets' potential to harbor and transmit AMR to their owners, and to identify most effective methods to increase risk awareness in consumers as well as intervention strategies promoting consumer behaviors to mitigate AMR transmissions at the human-animal interface.

## Introduction

In recent decades, antimicrobial resistance (AMR) has become a major global threat to public health, resulting in higher mortality, and prolonged illness in both human and animal populations (1). The acquisition of AMR in bacteria can occur either through gene mutation in the chromosomes of the organism itself or be caused by the exchange of mobile genetic elements with another organism (2). The horizontal transmission of mobile genetic elements between bacteria is of particular relevance, as it occurs independently of selective pressure caused by the presence of antimicrobial substances i.e., antimicrobials (AM).

Emerging AMR bacteria or resistance genes are released into the environment through body fluids and excretions of animals and humans; they can therefore be found in a variety of environmental compartments. Waste water or manure containing AMR determinants can contaminate fruit or vegetables crops, which then enter the supply chain for consumers, as well as the drinking water supply (3–5). In addition, evidence of direct transmission of AMR bacteria from livestock to farmers [e.g., (6–11)], from pets to their owners (12, 13) and/or *vice versa* have been described. Another potential source of AMR for humans is mishandled raw meat, which can be contaminated with AMR determinants due to cross-contamination either during the slaughtering of the animal or later during the processing of the meat. If contaminated raw meat is handled with insufficient hygiene measures, for example during meal preparation in the kitchen, AMR genes can be transferred to the consumer (14–16).

Hence, the general population is an important recipient of potential AMR determinants, whereby the pathways are diverse and involve a variety of sources. Highly complex graphs, so-called “confusograms,” have been published to illustrate AMR transmission pathways between different compartments [e.g., Department of Health, England, 2014; in other studies, schematic diagrams were used e.g., (4)]. However, although the links between compartments have been identified and described, the relative contribution of each compartment and pathway to the overall acquisition of AMR in humans remains largely unknown (4, 17).

The need to quantify the burden of different AMR transmission pathways with regard to their relevance to humans has been recognized (18). To mitigate the spread of such AMR bacteria or determinants, the most important sources and transmission pathways need to be identified. Moreover, an understanding of how consumers perceive and behave around these transmission pathways is needed.

Consumers play an important role in slowing down the spread of AMR by engaging in behaviors that prevent the transmission of AMR bacteria, such as practicing proper hygiene (19). It also has been recognized that communication with consumers about AMR is challenging, due to the complexity of the topic (20). Overall, little research has been published on intervention strategies targeting consumers with the objective of preventing AMR infections and transmission (21). To develop such strategies, it is important to understand consumer perceptions of AMR and of exposure pathways, as well as their current behaviors (22).

Surveys in medical settings have revealed that consumer awareness of AMR and its consequences is generally low (23, 24). Moreover, little is known about consumer perceptions and personal behavior related to AMR transmission pathways at the human–animal interface, regarding both live animals and products of animal origin. Although consumers are highly concerned about general microbial food hazards such as Salmonella (25), they show little knowledge about correct food handling practices at home and little intention of reducing their exposure to such risks at home (26, 27). The fact that consumers consider themselves safer cooks in comparison to average cooks might be a reason for this observation (28). They seem to be affected by the so-called “optimism bias” in their personal kitchen practices (29). Safe food preparation is established by habitual cooking (28) and by the degree to which people believe they have control over the outcome (e.g., food poisoning) (30).

Little is known about consumer perceptions and current behavior related to AMR in pets. The majority of pet owners are neither aware of the risk factors for zoonotic infections, the mechanisms of transmission, nor specific measures to prevent transmission of AMR bacteria (31–33), even among households with individuals at increased risk for acquiring an infectious disease, such as the elderly and people with immunocompromising conditions (34). Pet owners have reported household practices that even increase the spread of zoonoses, such as allowing the pet to sleep in one's bed and allowing dogs to lick a child's face (35).

In this study, our overall goal was to define the content of an intervention study by identifying the AMR transmission pathways that could be addressed to most effectively reduce the transmission of AMR from animal sources to humans. For this purpose, a qualitative approach was applied where a broad range of views can be consolidated with a limited number of participants (36). This study focused on the transmission sources and pathways at the human–animal interface, that is, healthy consumer contact with animal sources either directly via contact with live animals or indirectly via food of animal origin.

This study was conducted in three consecutive steps: The first step aimed to describe and quantify, in the Swiss context, the relevant sources and pathways of AMR exposure from animals to humans using expert opinion (Study 1). The second step investigated Swiss consumer perceptions and behavior regarding AMR and AMR transmission pathways around foods and pets (Study 2). In the third step, the identified AMR sources and pathways (outcome of Study 1) were compared to the perceptions and behaviors of consumers (Study 2). In this way, differences between the expert assessments of the importance of exposure pathways and the perceived importance of these pathways among consumers were identified. The identification of such gaps will foster the development of contents for targeted intervention studies for consumers.

## Study 1: Expert Opinion Assessment

### Materials and Methods

**Figure 1:**
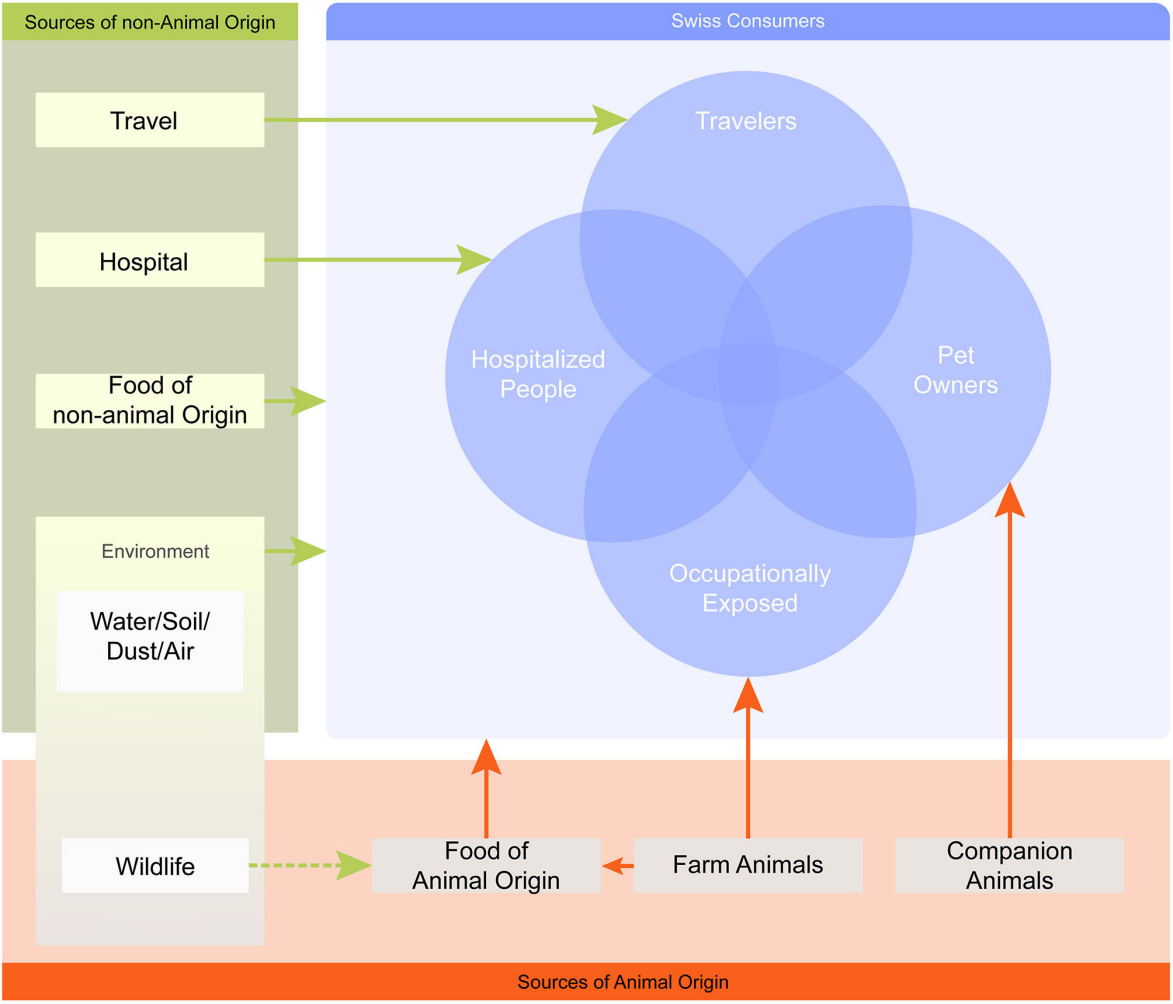
Map of AMR sources and transmission pathways relevant for the Swiss population. The map is simplified and only one-way transmission pathways to humans are considered, illustrated by arrows. The arrows link each AMR source with its relevant population (i.e., Swiss consumers) or specific subpopulation (e.g., pet owners), but do not inform about the nature of the interaction. The overlapping circles illustrate that an individual may be exposed to multiple sources at the same time.

A literature search was conducted to collect quantitative information on the prevalence of AMR hazards originating from different animal sources and evidence for their transmission to humans. Because of the very broad research field (no selection of a specific AMR determinant and consideration of many different source compartments), the relevant literature reviews were screened in a preliminary step and the relevant quantitative data was extracted. Whenever available, Swiss data was collected. If Swiss data was lacking, international research data was considered. For the purpose of this investigation, only transmission pathways from animal sources to humans were considered and not vice versa. The pathways assessed included direct transmission via animal–human contact and indirect contact through the exposure to food of animal origin. Also, we included fresh produce as food of non-animal origin since a contamination from animal sources is likely (e.g., through contaminated irrigation water or the spreading of manure). Moreover, the inclusion of fresh produce allowed the coverage of a wider spectrum of foods of relevance to consumers. Milk was excluded from the assessment, since raw milk is a negligible source of AMR hazards on the Swiss market. The main relevant AMR sources and transmission pathways for Swiss consumers and specific subpopulations are shown in Figure 1. The sources and subpopulations considered for this study are listed in Table 1.

**Table 1:** Subpopulations considered to assess the relative importance of antimicrobial resistance exposure pathways at the human-animal interface in the Swiss population.

**Subpopulations within the Swiss population**	**Exposure pathway(s) relevant to the subpopulations**	**Source**
Pet owners	Direct contact with animal/Indirect contact within household	Cats and dogs
Farmers	Direct contact with animal/Exposure via environment (manure, dust)	Livestock
Veterinarians	Direct contact	Pets and livestock
Consumers	Food preparation/Food consumption	Food of animal and non-animal origin

Following the literature review, an expert workshop was organized to assess the relative relevance of selected AMR transmission pathways (September 2018). Thirteen researchers involved in projects funded as part of the National Research Program “Antimicrobial Resistance” (NRP 72, www.nrp72.ch) of the Swiss National Science Foundation (SNF) were selected and invited to the workshop. Of these, seven participated in the workshop. The experts' main fields of expertise regarding AMR included small animal medicine (*n* = 1), ruminant medicine (*n* = 1), environmental sciences (*n* = 1), food safety sciences (*n* = 2), human medicine (1) and veterinary public health (*n* = 1). Two additional experts could not attend the workshop and were interviewed in person or through an online phone call prior to the workshop (expertise in hospital hygiene and vegetable plants).

The workshop followed the risk assessment framework of the World Organization for Animal Health (OIE) applicable to AMR hazards originating from animal sources (37). It was structured according to the following steps: hazard identification and release-, exposure- and consequence assessment. Because the present investigation focused on the transmission pathways of AMR genes (hazard) up to the consumer exposure, no consequence assessment was performed.

#### Hazard Identification

Our study did not focus on any specific AMR microorganisms or genes; rather, it aimed at considering the overall abundance of AMR as a hazard. The hazard was thus defined as an “AMR hazard,” which comprised both microorganisms and determinants to which resistance is expressed. AMR in non-pathogenic bacteria was also considered, whereas intrinsic AMR was excluded.

#### Release Assessment

The release assessment considered the abundance of AMR hazards originating from a specific source and thus considered the prevalence of different resistant bacteria or AMR determinants from identified sources.

#### Exposure Assessment

The exposure assessment considered both the recipients' intensity of contact with a specific source and the likelihood of AMR transmission. It thus assessed the likelihood of transmission of AMR hazards from a specified source to humans depending on the intensity of contact with the specific source, given that an AMR hazard is present. The exposure assessment did not consider the frequency of exposure to a specific source, but it focused on the likelihood of transmission during a contact.

A summary of the most relevant data published in regard to the prevalence of AMR hazards and their transmission from different sources to humans in a Swiss context was presented to the experts as a basis for discussion among them. The release assessment was then performed interactively during the workshop. Using visualization material, a vertical scale representing the prevalence of AMR hazards was displayed on a pin board, labeled from “very low” to “very high.” In a step-by-step procedure, the different sources were placed on the scale considering the evidence from the literature and modifications from the plenary discussion. Discussion was stopped when a joint agreement of the experts regarding the relative positioning of sources was reached. The focus of the position of different sources was put on the relative position between different sources rather than the absolute position on the scale. The same procedure was then repeated for the exposure assessment.

To estimate the frequency of exposure to AMR hazards for the population, person days at risk were calculated for relevant subpopulations in Switzerland. Person days at risk were calculated by multiplying the number of exposed people in the subpopulations by the number of days exposed to the respective source per year. The number of exposed people was mainly derived from Swiss national statics data, and the number of exposure days per year were estimated by the authors and verified in the framework of the expert workshop. All formulas, references and calculations for the generation of person days at risk are provided in the Supplementary Materials S1–S3. The relative contribution of different types of raw meat and fish was calculated based on Swiss consumption data (38).

## Results and Discussion

During the expert workshop, the experts suggested to limit the assessment to AMR hazards that are clinically relevant for humans in order to facilitate the ranking process. However, the present study aimed to avoid this limitation to AMR hazards where only single relevant bacteria or resistance determinant is taken into account. Special emphasis was thus placed on consideration of the comprehensive scope of AMR. Despite all efforts toward an unbiased assessment, it was recognized that the expert judgement was still likely to be biased toward clinically important AMR hazards, as most of the experts' daily work focused on clinically relevant AMR hazards such as extended-spectrum beta lactamase (ESBL) producers or methicillin-resistant *Staphylococcus aureus* (MRSA).

We also acknowledge that only a limited number of experts contributed to the study, however, all relevant disciplines were covered by at least one expert. A number of experts worked in multidisciplinary environments and were thus able to provide inputs for several compartments. It also needs to be considered that we did not aim to capture individual opinions, but rather stimulate the discussion within the group and toward finding the best agreement.

**Figure 2:**
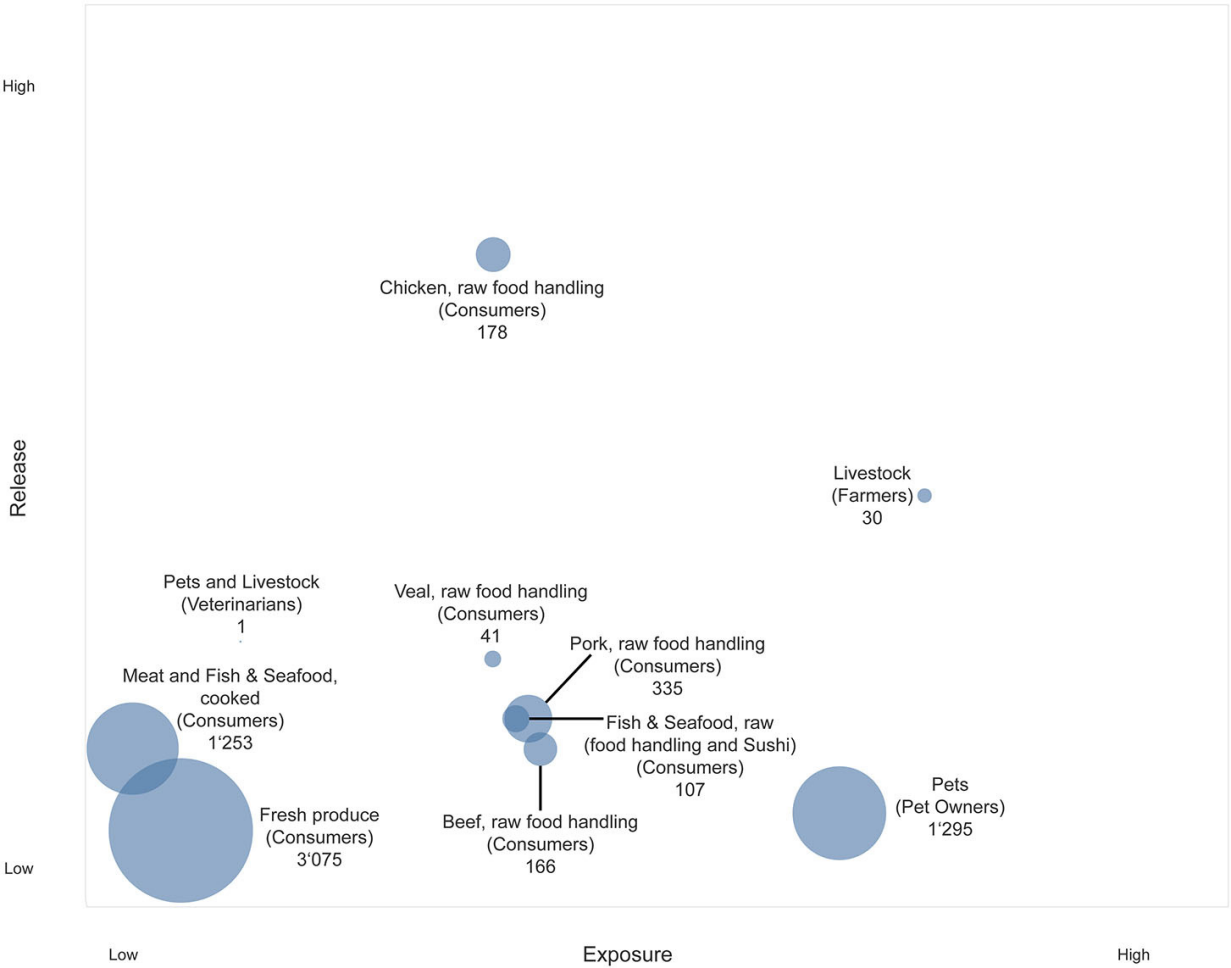
Bubble chart representing the exposure of the Swiss population to AMR hazards originating from food and animal sources. Y-axis: Release of AMR hazards from different sources. X-axis: Exposure to sources of AMR hazards for different subpopulations. Bubble size: Person days at risk [million days per year].

Considering the final output (Figure 2), release and exposure combinations of sources located toward the top right corner of the chart are of special interest, as they represent both high release of AMR in the source and high exposure of humans. In combination with a high number of person days at risk, sources located toward the top right corner of the chart are highly relevant for the Swiss population. The resulting person days at risk and the underlying assumptions for the different subpopulations are presented in Table 2. All numbers and sources of information for the calculation of exposed people are provided in the Supplementary Material S1–S3 (39–44).

**Table 2:** Person days at risk of exposure to antimicrobial resistant hazards at the human-animal interface in the Swiss population.

**Exposed subpopulation**	Number of people (*n*)^*^	**Assumed frequency of exposure (number of days per week)**	**Person days at risk (Mio. days per year)**
**SUBPOPULATIONS EXPOSED VIA DIRECT CONTACT WITH ANIMALS**			
Pet owners	3,547,930	Daily (7/7)	1,295
Livestock farmers	82,849	Daily (7/7)	30
Veterinarians (Clinicians)	2,550	80% employment (4/7)	0.5
**CONSUMERS EXPOSED VIA INDIRECT CONTACT (FOOD OF ANIMAL AND NON-ANIMAL ORIGIN)**			
Fresh produce	8,423,815	Daily (7/7)	3,075
Meat and fish/seafood COOKED	8,011,048	3 times per week (3/7)	1,253
Raw meat handling	8,011,048	2/3 of meat meals self-prepared	827
Chicken			178
Beef			166
Pork			335
Veal			41
Fish & Seafood			107

* *Numbers and sources of information for the calculation of exposed people are provided in the Supplementary Material S1–S3*.

The most distinct result was obtained for poultry meat, which ranked the highest by far according to the experts in terms of release. This was not surprising, and uncertainty among experts was therefore low. In contrast to the release, the uncertainty expressed by experts was higher in terms of exposure through poultry meat, which was also the case for other raw meat sources. Raw poultry meat ranked lower in the end than other meat sources on the exposure scale, because it was expected that consumer awareness regarding hygienic measure was higher for poultry meat than for other type of foods, and most likely lowest for fresh produce. One reason for the assumed high public awareness may be various nation-wide food safety campaigns launched in the past (for example, the campaign by the Food Safety and Veterinary Office (FSVO) “Richtig zubereiten—sicher geniessen” [“Proper preparation—safe enjoyment”], www.sichergeniessen.ch). For fresh produce, both the release and the exposure were considered low, whereas the person days at risk for this source were highest.

Farmers ranked highest in terms of human exposure, which is understood as the probability of transmission of AMR hazards from the source. Arguments to justify this high position were the close and sustained contact between livestock and farmers and the additional burden through substantial environmental contamination, such as the exposure through dust. Following this argument, veterinarians were ranked much lower in terms of exposure, because the application of more stringent sanitary measures was assumed.

Although pets ranked low as a source of AMR release, the exposure of pet owners was assessed as high because of the presumed close contact between pets and their owners. However, the experts' uncertainty regarding the position of the pets in the overall framework was rather high. Furthermore, experts were at odds regarding the number of days at risk for pet owners. There was a debate regarding the extent to which a pet could actually pose a risk as an AMR reservoir to its owner while not being subject to antimicrobial treatment. Only a few studies have investigated the existence of AMR hazards in healthy dog populations, however most of them focused on a specific AMR hazard [e.g., (12, 45, 46)] and would thus not capture the whole range of AMR bacteria or determinants presents. Overall, the experts recognized that the transmission of AMR from companion animals to humans is complex and requires further investigation.

Although not being part of the final framework, the experts discussed the burden of the environmental compartment. Again, the uncertainty was very high in terms of both release and exposure. While a number of publications address AMR in the context of farmland and food production, literature on AMR determinants in natural environments seems to be very limited. Even if there was substantial release from the environmental compartments such as soil or surface water, it was assumed that exposure during recreational activities such as hiking or biking would be low. The only relevant exposure mentioned was the direct intake of surface water, which might pose a certain risk for swimmers or scuba divers.

A general limitation inherent to our assessment was that we disregarded simultaneous exposure to multiple pathways, and thus the potential accumulation of exposure to AMR sources (for example owning a pet and handling raw meat). Furthermore, this study only considered the release and exposure assessments of the different pathways, but not their consequences on the human side. The assessment thus does not take into account the clinical relevance for the exposed individual. Therefore, validity is only given for the specific purpose of this assessment, which was the overall exposure of humans to AMR hazards from different animal and food sources.

## Study 2: Consumer Perception and Behaviors

The objective of Study 2 was to uncover the potential perceptions that consumers (i.e., food preparers and pet owners) have about AMR in general and about the possible exposure pathways. Furthermore, the study aimed to assess consumers' food- and pet-related behaviors in terms of hygiene.

### Materials and Methods

#### Sample

Participants were invited for an interview on safe food handling or safe pet handling. They were recruited through online portals and advertisements in supermarkets in the German-speaking part of Switzerland. The inclusion criteria for interview participation were a high level of spoken German, a minimum age of 18 years and either food preparation at home at least two times a week or the ownership of a pet (preferably cat or dog). A limitation is therefore, that the current study was focused on consumers from a specific part of Switzerland only. Each participant was interviewed on both transmission pathways of food and pets. Although the sample size was small, care was taken that the participants covered various characteristics of the consumer population (e.g., consumers of different ages, gender and household types). Seven males and seven females participated in the interviews, ages ranging from 23 to 63 years. Recruiting and interviewing continued until saturation had emerged after the fourteenth participant, i.e., when no new information was obtained from the interviews. Saturation is a common and established stopping rule in qualitative interview studies (47). Although the small number of interviews may not be representative of the Swiss population, it sufficed to provide an impression of issues that can play a role in consumer perceptions and behaviors related to AMR. Potential interviewees were screened via phone call to determine whether they were eligible to participate.

#### Procedure

The interviews took place at the participants' homes between June and August 2018, lasted from 45 to 60 min and were carried out in German. All interviews were conducted by the same person. Participants were only informed about the broad topic of the interview, i.e., “safe food handling” or “safe pet handling.” The interviewer assured anonymity and confidential treatment of the data and obtained approval to tape the interview. The interview was only conducted after the participant had provided written informed consent. Interviews followed a guideline. At the end of the interview, the purpose of the study was explained in detail to the participants and they could ask questions. They then received 50 CHF as compensation.

#### Materials

Based on a literature review and team discussions, two interview guidelines were developed: one started with the topic food preparation and one with the topic pet care (Supplementary Materials S4, S5). The interview guidelines were piloted on three personal contacts that fulfilled the inclusion criteria prior to the actual data collection. The guidelines covered four main topics: (1) interviewees' perceived risks of foods or pets in general; (2) their awareness, knowledge, beliefs and perceived risks of AMR; (3) their perception of personal exposure pathways (food or pet), as well as of other possible transmission pathways of AMR; and (4) possible measures to mitigate the risks of the exposure pathways and current preventive behavior. Additionally, pet owners were asked about their attitude regarding AM treatments for their pets.

#### Analyses

Interviews were transcribed using the F4 transcription software (f4transkript, Marburg, 2018), and coded and analyzed using the MAXQDA software (VERBI Software, Berlin, 2018). The codes were developed based on the interview guidelines. Additional codes were identified while reading the interviews. All interviews were coded by the same researcher. Additionally, six interviews were coded by a second coder and then compared with the first coder. In case of conflict, the two coders discussed their findings and sought a compromise.

## Results and Discussion

### Awareness of Hazards From Food and Pet Handling

Overall, interviewees were not aware that AMR can be contracted through food products or close contact with pets. Food preparers perceived genetically modified foods, food additives (e.g., E-numbers and flavor enhancers) and the use of agricultural pesticides (e.g., glyphosate) as the main health hazards related to food products. Food poisoning and stomach problems were also quoted but without noting bacteria, let alone AMR. Pet owner concerns referred to overtaxing, poor quality pet training, injuries from bites, and scratches and allergies. Only a few interviewees mentioned the possibility of pathogen transmission from pets to humans such as tick-borne diseases or infections with tapeworms, but did not specifically refer to AMR bacteria. The increased awareness of these hazards might result from the fact that they are more frequently addressed by veterinarians.

*I don't know, if an illness can be an issue. I didn't hear about it often. I mean an infection from animals to humans. A few things exist, but I don't think it is a big danger.”* [Assessment of hazards through pets] (pet owner, 50–59 years old).

### Knowledge Gaps

The majority of participants were familiar with the existence of AMR, the emergence of AMR in humans through the use of AM and possible negative consequences such as an increased risk of a longer course of disease.

*Well, antibiotics are effective against bacteria. It means antibiotics won't be effective anymore. It can't be used any longer for an illness usually treatable with it. In worst case scenario, it can be life-threatening*. [Meaning of AMR] (pet owner, 40–49 years old)

Furthermore, some participants mentioned that risks of AMR are particularly high for those with a compromised or incompletely developed immune system, such as the young, elderly, pregnant, and those with immune function-reducing conditions.

Substantial knowledge gaps emerged regarding the transmission pathways of AMR. Interviewees did not feel well-informed about the topic. They believed that only the use of AM results in resistance, without being aware of the fact that AMR can spread between different sources. Public places (e.g., toilets and buses), places in nature (e.g., waters, forests and air) and the exchange of body fluids (e.g., through sexual intercourse or syringes) were mentioned as known transmission routes. Contact through feces, skin, hair, salvia, blood, or other fluids were reported as possible exposure routes for AMR between animals and humans. Respondents did not distinguish between the various types of infectious agents (viruses, bacteria, fungi, etc.).

We also identified some confusion regarding the term bacteria. First, most participants did not differentiate between the transmission of AMR bacteria and bacteria not resistant to antibiotics. Their one-dimensional concept of bacteria included health-preserving bacteria, harmful bacteria and AMR bacteria without differentiating between their various sources and consequences. Some participants emphasized the important role of bacteria in our bodies. At the same time, the source of AMR bacteria was frequently associated with dirt.

*I think bacteria are on dirty places and a lot of dirt is outside*. [Assessment of transmission pathways] (food preparer, 20–29 years old)

Second, most participants assumed that carrying AMR bacteria would always cause physical symptoms and thus be noticed. Third, a number of participants believed that AM are effective against bacteria and viruses, which consequently leads to a misconception with regard to the biological mechanisms underlying the spread of AMR.

### Risk Perception

Participants' understanding of the risks of AMR was mostly based on their personal experiences. Very few of the interviewees had previous experience with AMR bacteria causing a life-threatening infection among family members, friends or colleagues, so they perceived little risk in AMR. In line with the optimism bias (29, 48), the majority of the interviewees did not believe they were personally at risk, but acknowledged that AMR could constitute a danger to society. Similarly, the risk of foreign and conventionally produced food products was perceived to be higher compared to Swiss and organic products. Their great trust in Swiss food products was reasoned by the strict Swiss food legislation and consequent hygiene controls.

*Everything has to be declared in Switzerland, and we have the choice what we want to eat. Thus, the risk is relatively low here. It is quite safe to buy food in Switzerland. My biggest concern abroad is to get a bacterial infection*. [Assessment of food products with an AMR risk] (food preparer, 30–39 years old)

Most participants viewed animal products, especially meat, as more susceptible to AMR than products of non-animal origin. Some explained this by the use of AM in livestock farming. Others believed this because they either knew these foods belong in the fridge or that protein foods from animals constitute a high risk for food poisoning.

Some participants emphasized that chicken and eggs are risky, since these products are known for carrying food poisoning bacteria such as *Salmonella*. *Campylobacter* was not mentioned, although it is the most common food-related zoonotic agent causing diarrhea in Switzerland (49). Fish and seafood may be less salient, since Swiss people consume it less than meat (49). Participants were uncertain regarding the risk originating from vegetables and fruits, as well as from industrially produced food products and convenience foods.

Overall, pet owners did not perceive their own companion animal as a risk for AMR transmission, or generally as a source of zoonotic diseases. This can also be explained by the optimism bias [see above as well as Sutton (48) and Weinstein (29)]; other pets and other owners were perceived as being at greater risk than the interviewee. Although pet owners were aware of the existence of zoonotic diseases, they did not expect it amongst Swiss pets. An explanation might be that people perceive less risk if it is chosen voluntarily (50). Furthermore, some participants mentioned that animals help strengthen children's immune systems and therefore protect them from illnesses and allergies. People are known to tolerate higher risks from activities seen as highly beneficial (51). Cats and dogs were more frequently considered as possible AMR carriers than other pets because of the close contact to their owners and the frequent contacts with other animals outdoors.

All respondents emphasized the risks of AMR transmissions abroad. They assumed people living in third world countries to be at greater risk for AMR, since these countries are associated with poor hygiene. Industrialized nations with a high consumption of AM in intensive livestock farming were also considered to pose a higher risk than Switzerland. Also, participants who mentioned hospitals as a source of AMR emphasized that hospitals abroad were of greater concern than Swiss hospitals. Public media are consumers' main information source about AMR, which may give the impression that dirty hospitals are the main source (52).

*Pork and chicken from factory farming. In Hungary, Poland, Germany, partially Belgium, which all have completely different animal welfare laws and an extreme use of antibiotics. In these cases, it depends if you cook the meat properly. I don't know about vegetables*. [Assessment of food products with an AMR risk] (food preparer, 30–39 years old)

The fact that interviewees rated traveling abroad as more risky than food products or pets can be explained by people's unfamiliarity with foreign regions. Risk perception is related to factors such as newness, controllability or observability, whereby unfamiliar and dreadful hazards are perceived as most risky (50). Furthermore, food poisoning might be more frequently portrayed in the media as a risk abroad (53).

### Awareness of Preventive Measures

At the beginning of the interview, without referring to AMR, food preparers were asked to describe measures that they knew related to the keywords *clean, separate, cook* and *chill*, as recommended by the FSVO (54). Results showed that hygienic food handling measures related to *clean* were more prevalent and salient in participants' minds, such as cleaning food products and handwashing prior to preparation and consumption. Their motivation to clean food products, mostly fruits and vegetables, was to remove dirt and pesticides rather than AMR bacteria.

Most participants did not know which measures were meant by the recommendation *separate*. Only a few reported keeping raw poultry or meat separate from ready-to-eat food products.

*I do not care about separating. I put everything in the refrigerator and that is it. OK, maybe not with already opened food products. We store it in a food container or wrap it up so we do not have poultry blood in the fridge. But we do not separate it in a way that we have one compartment only for meat or dairy products* (food preparer, 20–29 years old).

Regarding *chill*, some interviewees knew that a certain temperature for refrigerators is recommended without knowing the exact degrees. They were not aware that refrigeration slows down bacterial growth. Spoilage of food was associated more strongly with mold than bacteria.

Concerning *cook* measures, participants were aware of concerns regarding undercooked meat because “something” could happen, but not everybody knew that the goal of heating food to a high temperature is to destroy active bacteria. Nobody reported validating the accuracy of the meat's temperature with a thermometer.

Later in the interview, food preparers were asked about measures to prevent AMR transmission in the kitchen (Supplementary Material S6). Most of the reported measures were general hygiene measures. Interviewees were thus either aware that proper kitchen hygiene constitutes a prevention tool against food-borne illnesses and therefore concluded that these are also effective for the prevention of AMR hazards or these measures were salient, as kitchen hygiene practices had already been discussed at the beginning of the interview (55). As preventive behavior to mitigate AMR exposure, pet owners most frequently mentioned general measures that promote the animals' health (Supplementary Material S7). Further suggestions were to restrict close contact with other animals.

*Avoiding close contact with other animals*. *Or even with humans, but I don't want this. Paying attention to what my dog eats outside. I could wash him, or maybe go to the veterinarian regularly*. [Measures to avoid AMR on pets] (pet owner, 30–39 years old)

Surprisingly, some pet owners did not know whether medicine prescribed for their animal in the past was antibiotics or something else. Probably as a result of their low problem awareness, they had not asked the veterinarian about the prescribed treatment.

A disparity was apparent in our sample between participants' knowledge of possible hygiene measures and self-reported practices (Supplementary Materials S6, S7). The awareness of such strategies thus does not mean that respondents put them into practice (56). On the contrary, pet owners reported personal behaviors that may promote the transmission of AMR between pets and family members. For instance, most pets had their food bowls in the kitchen, and some dogs and cats were allowed to sleep in the pet owner's bed.

### Psychosocial Determinants of AMR-Preventive Behavior

All participants mentioned a lack of knowledge about AMR and preventive measures as the main barrier preventing them from carrying out food or pet safety actions. Since knowledge gaps about AM and AMR have been found to be a critical determinant of non-adherence to physicians' instructions (57), they might also be an important determinant of non-adherence to preventive measures. Consumers need to understand that AMR can spread between different sources, independent of the consumption of AM. In addition, they need to be informed of the measures that prevent such transmissions. Participants seemed unaware of the antibacterial effect of some hygienic behavior in the kitchen or among pets. They anticipated hygiene measures with negative consequences such as being time-consuming rather than with positive outcomes. Additionally, the implementation of preventive measures was considered as unnecessary in a highly developed country such as Switzerland. In other words, they had low outcome expectations (58) regarding the benefits of hygiene measures.

Our data also indicated there was a low sense of personal ability to help to contain the problem because infections were perceived as outside the influence of individuals. People who perceive that conditions are due to forces beyond their control tend to take more risks (59), which might further explain why our participants did not adopt safety measures against transmissions. Specifically, pet owners perceived low control over the transmission of AMR to their pet, or over the transmission from their pet to themselves. That is, they exhibited low perceived behavioral control (60, 61).

*I can't control whether my cat comes back home with AMR or not. What can I do about it?* [Measures to avoid AMR on pets] (pet owner, 20–29 years old).

Even if some respondents recognized AMR as a problem in the community, most did not feel they had a personal role in either the problem or the solution. Interviewees believed that improving and maintaining food safety should be achieved mainly by farmers and food producers higher in the food supply chain and prior to food offered for sale, which is a denial of responsibility (62). The fact that food preparers are not aware that the majority of food poisoning cases originate at home may explain this (63).

## General Discussion

### Comparison Experts vs. Consumers

Three important differences regarding risk estimates of meat products emerged between experts and consumers. First, experts clearly distinguished between raw and processed meat in their risk estimates as the risk of contaminated meat is reduced by cooking or other modern sanitary techniques to kill pathogenic germs (64). Therefore, experts assumed a potential AMR transmission risk for raw food products only. On the other hand, most consumers did not distinguish between raw and processed meat, which might be a reason why safety practices such as cooking were not considered as preventive measures against AMR.

Second, experts rated the *release* of AMR hazards much higher for raw poultry than for raw red meat (i.e., beef, pork and veal), raw fish or raw seafood, while they rated the *exposure* risk as the same for these food products. Experts namely expected that lay people's awareness regarding hygienic measures is higher for raw poultry meat than for other types of raw meat. However, most lay people underestimated the risk of raw poultry meat compared to other raw animal food products. Thus, they do not implement preventive measures as expected or recommended by experts (54). Generally, experts assumed the AMR transmission risk through food products to be low, as long as hygienic kitchen practices are implemented. Yet, lay people were not aware of all recommended preventive measures, let alone adopted them in their daily lives.

Third, lay people believed that organic and Swiss-produced meat is not affected or is less affected by AMR, a common misconception in consumers (65). They should therefore be informed that AMR bacteria can be present on foods independent of their origin and production system.

Regarding pets as an exposure source, experts acknowledged research showing that animals may act as reservoirs for resistant organisms, especially during acute infection or AM treatment. However, as mentioned above, experts regarded the uncertainty about the AMR *release* from healthy pets to be high. The *exposure* for pet owners was assessed as rather high, because of the presumed close physical contact of pets and their owners. The interview study confirmed that pet owners were in close contact with their pets but interviewees' risk perception regarding the AMR release by their own pets was extremely low. Contrary to transmission pathways at the human–animal interface, lay people generally perceived the AMR risk through the environment to have a greater impact than animal sources. In comparison, experts assessed the risk of AMR contraction for recreationally active people due to the release of AMR from the environment or surface water to be very low.

The gaps between experts' and consumers' assessments might in part have emerged due to the different assessment criteria used to rank various AMR transmission pathways (66). Although interviewees were aware that certain sources may release AMR bacteria, they did not include the magnitude of the release in their risk assessments. Previous research has shown that lay people tend to rate a risk based on their exposure to a hazard, without considering the release as experts do (67). This was confirmed in our study, as interviewees based their assessment on their personal exposure to a certain source or else on the size of the subpopulation (e.g., more people were food preparers than pet owners), while experts combined all factors for their ranking in the workshop. Therefore, different conceptions, assumptions, and values might underlie much of the discrepancy between experts and lay peoples' risk ranking.

### Implications for Practice and Future Research

The ultimate aim of this paper was to identify the relevant content and direction for an intervention aimed at consumers to reduce their exposure to AMR hazards from animals and food sources. Although the present results are linked to the Swiss context, they may be transferred to other regions that have a comparable AMR situation, consumer culture and food safety standards. Taking into consideration the specific conditions such as prevalence of AMR in different sources, relevant subpopulations and food and pet handling habits of the target population, the assessment can also be applied in a different geographical and cultural context.

In the present context, practical implications could be gained from the comparison between expert opinions and lay peoples' perceptions of different AMR exposure pathways. Interventions aimed at food preparers should address the potential risk of AMR hazards in raw meat. Specifically, raw poultry meat should be emphasized as risky because of its high AMR release, and because consumers underestimate the increased risk of pathogens in poultry meat. Additionally, it is important to communicate that organic and local meat products are also potential AMR sources [e.g., Garcia and Teixeira (65), although Switzerland has high food safety and quality standards (68)].

We also recommend educating lay people about these facts in order to raise their awareness about the spread of AMR (69). Providing risk information (e.g., AMR prevalence in raw poultry meat) can further increase their risk perception of AMR (70). Interventions should further include instructions on how to implement preventive measures and information about the antibacterial outcomes of these hygiene practices. Advice on how consumers may overcome barriers to preventive behavior (e.g., habits or lack of time) should also be addressed. Planning the implementation of preventive measures in everyday life may serve as a tool to ensure that people not only intend to implement these measures, but actually execute them (71).

To date, quantitative data about the prevalence of AMR in pets and the likelihood of transmission to their owners or veterinarians is scarce. However, taking into account the large number of pet owners in Switzerland (3.5 million; see Table 2), this matter deserves our attention. More research is indeed needed to assess the release of AMR bacteria and its exposure from pets to humans, so that evidence-based recommendations for preventive measures can be made. Interventions targeting pet owners should primarily enhance their risk awareness about pets as a potential source of pathogenic microorganisms, which include AMR bacteria. Pet owners would notably benefit from clear and practical safety recommendations, similar to the “*clean, separate, cook and chill*” recommendations for food preparation. In addition, quantitative studies are needed to indicate the psychosocial determinants underlying pet owners' preventive behavior. Interventions can then be targeted toward these determinants in order to reduce pet owner exposure to potentially harmful AMR or other microbiological hazards originating from their pets.

To conclude, awareness about AMR transmission pathways through food and animals should be increased among lay people and common misconceptions be overcome. Additionally, further investigations are needed to quantify the release of AMR in pets and the likelihood of transmission to their owners.

## Data Availability Statement

The raw data supporting the conclusions of this article will be made available by the authors, without undue reservation.

## Ethics Statement

Ethical review and approval was not required for the study on human participants in accordance with the local legislation and institutional requirements. Written informed consent for participation was not required for this study in accordance with the national legislation and the institutional requirements.

## Author Contributions

IL and CF designed, collected and analyzed the data, and drafted the manuscript. VV and KS supervised the project work, contributed to the concept and design of the study, and revised the manuscript. VV acquired the funds. All authors contributed to the article and approved the submitted version.

## Conflict of Interest

IL and KS were employed by the company SAFOSO AG. The remaining authors declare that the research was conducted in the absence of any commercial or financial relationships that could be construed as a potential conflict of interest.
