# Supplementary material for: Exposure Pathways to Antimicrobial Resistance at the Human-Animal Interface—A Qualitative Comparison of Swiss Expert and Consumer Opinions
Source: Front Public Health. 2020 Jul 30;8:345. doi: 10.3389/fpubh.2020.00345 (PMC7406637; doi:10.3389/fpubh.2020.00345)
Supplement: Supplementary file 1 [file Data_Sheet_1.docx]

**S1****. Input numbers and references for the calculations of person days-at-risk for different target groups in the Swiss population exposed to AMR**

Person days at risk per year (PDAR) = Number of exposed people in the population (E) × Estimated frequency of exposure days per year (F)

PDAR = E × F

Formulas and input numbers for calculations are provided in S2 and S3.

Results were based on non-rounded input numbers. For the purpose of readability, all input values are rounded to whole numbers.

The references provided below are applicable for estimating the PDAR for the Swiss population. The equations are kept generic to assure applicability to other populations.

**S2. Formulas, input numbers and references for the calculation of person days-at-risk (PDAR) for different target groups in the Swiss population exposed to AMR**

| **Target Group** | **Number of people exposed (E)** | | **Number of exposure days per year (F)** | | **Total PDAR** |
| --- | --- | --- | --- | --- | --- |
|  | **Formula and References for Switzerland** | **Calculations for Switzerland** | **Estimation by experts** | **Days/year** | **E × F** |
| Pet Owners | [number of private households]^*^ ×  [% of households with at least on pet]^†^ × [average number of persons per household]^*^ | 3,700,000 × 2.23 × 0.43 = 3,547,930 | 7/7 days per week | 365 | 3,547,930 × 365 = 1,294,994,450 |
| Livestock Famers | [number of farming businesses in the animal production sector]^‡^ × [average number of persons per household]^*^ | 37,152 × 2.23 = 82,849 | 7/7 days per week | 365 | 82,849 × 365 = 30,239,870 |
| Veterinarians | Number of Veterinarians^§^ | 2,550 | 4/7 days per week | 209 | 2,550 × 209 = 531,857 |
| Fresh Produce | [Total population size]^**^– [number of babies ≤ 1 year]^††^ | 8,508,904 - 85,089 = 8,423,815 | 7/7 days per week | 365 | 8,423,815 × 365 = 3,074,692,460 |
| Consumers of meat (cooked) | [Total population size]^**^ – [number of babies ≤ 1 year]^††^ – [number of vegetarians]^‡‡^ | 8,508,904 - 85,089 -412,767 = 8,011,048 | 3/7 days per week | 156 | 8,011,048 × 156 = 1,253,156,799 |
| Consumers handling raw meat | [Total population size]^**^ – [number of babies ≤ 1 year] ^††^ – [number of vegetarians]^‡‡^ | 8,508,904 - 85,089 -412,767 = 8,011,048 | [Number of exposure days to meat] × [Percentage of days, when a hot meal is cooked] ^‡‡^ | 156 × 0.66 = 103 | 8,011,048 × 103 = 827,083,487 |

^*^ Federal Statistical Office (FSO). Population and Households Statistics STATPOP. https://www.bfs.admin.ch/bfs/en/home/statistics/population/effectif-change/households.html. Published 2018. Accessed December 17, 2018. [82]

^†^ Verband für Heimtiernahrung VHN. Statistik Heimtierpopulation 2018. https://www.vhn.ch/wp-content/uploads/2018/04/Statistik-VHN-Heimtierpopulation-2018.pdf. Published 2018. Accessed December 17, 2018. [83]

^‡^ Federal Statistical Office (FSO). *Food and Agriculture Pocket Statistics 2018*.; 2018. [84]

^§^ Personal Communication

^**^ Federal Statistical Office (FSO). Ständige Wohnbevölkerung nach Staatsangehörigkeitskategorie, Alter und Kanton, 2. Quartal 2018. Federal Statistical Office (FSO). [85]

^††^ Altersaufbau der Bevölkerung. https://www.bfs.admin.ch/bfs/de/home/statistiken/bevoelkerung/stand-entwicklung/alter-zivilstand-staatsangehoerigkeit.assetdetail.5887358.html. Published 2018. Accessed December 17, 2018. [86]

^‡‡^Weighted average of weekdays and weekends, from: Federal Office of Public Health (FOPH) and Federal Veterinary and Food Safety Office (FSVO). *Anthropometric characteristics and indicators of eating and physical activity behaviors in the Swiss adult population. Results from menuCH 2014-2015*.; 2017. [87]

**S3. Relative contribution of different types of meat and fish to person days at risk (PDAR) for Swiss consumers.**

| **Meat type** | **Consumption (g/consumer/day)*** | **Relative Share** | **Total PDAR**  **(days per year)** |
| --- | --- | --- | --- |
| Pork | 62.38 | 41% | 335,436,369 |
| Poultry | 33.1 | 22% | 177,988,840 |
| Beef | 30.79 | 20% | 165,567,262 |
| Seafood + Fish | 19.95 | 13% | 107,277,261 |
| Veal | 7.59 | 5% | 40,813,755 |
| **Total** | **153.81** | **100%** | **827,083,487** |

* Jans C, Sarno E, Collineau L, Meile L, Stärk KDC, Stephan R. Consumer exposure to antimicrobial resistant bacteria from food at Swiss retail level. Front Microbiol. 2018;9(MAR). doi:10.3389/fmicb.2018.00362.

**S4. Interview guideline for food prepares**

1. Welcome the interviewee
2. Thank for participating
3. Instructions on the procedure (e.g. audio recording)
4. Time frame of approx. 1 hour
5. Emphasize voluntariness and anonymity
6. Written informed consent
7. Ask for demographic information:

- Diet (e.g. vegetarian)
- Frequency of food preparation at home
- Household composition (child/ren)
- Age
- Profession
- Pets

1. Ask whether the interviewee has any questions

| *Subtopic* | *General questions* | *Specific questions (if it was not answered by what the interviewee said)* |
| --- | --- | --- |
| Individual food handling | First, I would like to talk to you about food. Please feel free to tell me anything you can think of. These are all important insights for us.  What do you associate with "hazards through food consumption"?  *If not mentioned by the interviewee:* What comes to your mind about infectious diseases from foods?  What does «safe food handling» mean to you?  How do you ensure safe food handling at home to prevent infectious diseases?  When you buy food, what do you pay attention to with regard to bacteria?  Food safety authorities recommend the following food safety measures: heat, cool, clean, and separate. What preventive practices do you have in mind?  **Interviewees were asked to comment on images of safe food handling measures (separate, cook, chill, clean).** | What experiences have you already had with food poisoning?  What was the reason?  How could you have prevented it?  What does it prevent?  What foods are you handling that way and why?  What does it prevent?  What foods are you handling that way and why?  Why is it important?  Which of these measures aren`t you implementing at home and why?  Why should you implement these measures? |
| Antibiotic resistance transmission pathways | Antibiotic resistant bacteria can also be transmitted from foods to humans, if food is not handled properly.  What does "antibiotic-resistant bacteria" mean to you?  **Explain that bacteria become resistant and not the body.**  How do antibiotic resistant bacteria get into our food?  How likely is it that antibiotic resistant bacteria will be transferred to you via food?  Which other transmission possibilities do you know for a transmission of antibiotic resistant bacteria to humans?  *If not mentioned by the interviewee:* Antibiotic resistant bacteria can be transmitted from pets to humans. How are antibiotic resistant bacteria transmitted?  How do antibiotic resistant bacteria get to pets?  *If not mentioned by the interviewee:* Antibiotic resistant bacteria can also be transmitted to you while you are abroad. How can antibiotic resistant bacteria be transmitted when travelling abroad? (e.g. food, water, hospital visits)  Which of these sources (pet, food or travelling abroad) is most/least likely to transmit antibiotic resistant bacteria to humans? | How can bacteria become resistant?  Which foods are affected and why?  How likely is it that these bacteria will lead to negative consequences for you (e.g. health problems)?  How likely is this for other people?  Which people are often in contact with antibiotic resistant bacteria?  Which animals could be at risk for carrying antibiotic resistant bacteria and why?  How likely is it that antibiotic resistant bacteria are transmitted from pets to humans?  How likely is it that antibiotic resistant bacteria through pets will lead to serious consequences?  In which countries/regions is the antibiotic resistant bacteria risk high and why?  How likely is it to get antibiotic resistant bacteria abroad?  How likely is it to become seriously ill from antibiotic resistant bacteria abroad?  Which of these transmission possibilities might be most relevant for you?  When are negative consequences most likely? |
| Preventive measures  Behavioural barriers | What is the problem about antibiotic resistant bacteria and the spread of antibiotic resistant bacteria?  What can you do to protect yourself from antibiotic resistant bacteria?  *If not mentioned by the interviewee:* How can you prevent the spread of antibiotic resistant bacteria through food at home?  *If not mentioned by the interviewee:* How can you prevent the transmission of antibiotic resistant bacteria to yourself abroad?  What can pet owners do to prevent an antibiotic resistant bacteria transmission between them and their pet?  What reasons can you think of that could prevent people from implementing such behaviours when handling food?  What barriers could you think of for people dealing with pets?  What barriers could you think of when people are abroad? | What are the consequences of antibiotic resistant bacteria for people/animals?  Why is it helpful?  Which measures did you already implement and why?  Which measures aren`t you adopting and why?  What effect does (*mentioned behaviour*) have?  How difficult is it to implement (*mentioned behaviour*)?  Why is it difficult?  How effective are these measures?  What could be motivating?  What could be supportive? |
| Intervention to reduce antibiotic resistance | Do you intent to do anything against the spread of antibiotic resistant bacteria in the future?  What do you need to implement such measures?  Imagine an online intervention (e.g. website) is developed with the aim of promoting safe handling of food in order to reduce the transmission of antibiotic resistant bacteria. How should this intervention be designed?  **Interviewees had to make commentaries on ideas (e.g. facts or case study) on how to implement the intervention.**  What do you think of reminders of certain measures that can be easily integrated into everyday life? E.g. via the smartphone.  Have you recently seen a campaign on food consumption and bacteria?  Now the last question: Where did you hear about antibiotic resistant bacteria? | *If yes:* How will you implement this intention?  *If no:* Why not?  Which information could be useful?  Do you think consumers would us it?  Why would you use it?  Can you think of any other possibilities that could motivate people to change their behaviour?  What did you think about it?  Did you search for further information?  *If yes:* Why and where?  *If no:* Why not? |

**S5. Interview guideline for pet owners**

1. Welcome the interviewee
2. Thank for participating
3. Instructions on the procedure (e.g. audio recording)
4. Time frame of approx. 1 hour
5. Emphasize voluntariness and anonymity
6. Written informed consent
7. Ask for demographic information:

- Pets: How many? Which animals?
- Age of interviewee
- Household composition (child/ren)
- Profession
- Diet (e.g. vegetarian) and frequency of food preparation at home

1. Ask whether the interviewee has any questions

| *Subtopic* | | *General questions* | *Specific (if it was not answered by what the interviewee said)* |  |
| --- | --- | --- | --- | --- |
| Individual pet handling | | First, I would like to talk to you about your pets. Please feel free to tell me anything you can think of. These are all important insights for us.  What do you associate with "hazards for humans through pets"?  *If not mentioned by the interviewee:* What comes to your mind about infectious diseases between pets and owners/people?  What does «safe pet handling» mean to you?  How do you ensure safe pet handling at home to prevent infectious diseases?  What measures are recommended by experts to prevent the transmission of pathogens from pets to humans? | What experiences have you already had with zoonosis  What was the reason?  How could you have prevented it?  What does it prevent?  What foods are you handling that way and why?  Why is it important?  Which of these measures aren`t you implementing at home and why?  Why should you implement these measures? |  |
| Antibiotic resistance transmission pathways | Antibiotic resistant bacteria can also be transmitted from pets to humans.  What does "antibiotic-resistant bacteria" mean to you?  **Explain that bacteria become resistant and not the body.**  How are antibiotic resistant bacteria transmitted from pets to humans?  How likely is it that antibiotic resistant bacteria will be transmitted from your pets to you?  How do resistant bacteria get to pet(s)?  *If mentioned by the interviewee:* You mentioned antibiotics. What is your attitude towards antibiotic prescriptions for your pet?  Can you tell me about situations where your animal was sick and needed antibiotic treatment?  Which other possibilities do you know for the transmission of antibiotic resistant bacteria to your pet?  *If not mentioned by the interviewee:* Antibiotic resistant bacteria can also be transmitted to your pet by other animals or humans. How are antibiotic resistant bacteria transmitted?  *If not mentioned by the interviewee:* How likely is antibiotic resistant bacteria in pet food?  Which of these transmission possibilities might be most relevant for your pet? Why?  Which other transmission possibilities do you know for a transmission of antibiotic resistant bacteria to humans?  *If not mentioned by the interviewee:* Antibiotic resistant bacteria can also be transmitted to humans via food. How do antibiotic resistant bacteria get into food? (e.g. meat)  *If not mentioned by the interviewee:* antibiotic resistant bacteria can also be transmitted to you while you are abroad. How can antibiotic resistant bacteria be transmitted when travelling abroad? (e.g. food, water, hospital visits)  Which of these sources (pet, food or travelling abroad) is most/least likely to transmit antibiotic resistant bacteria to humans? | | How can bacteria become resistant?  How likely is it that these bacteria will lead to negative consequences for you (e.g. health problems)?  How likely is this for other people?  Which people are often in contact with antibiotic resistant bacteria?  Which animals could be at risk for carrying antibiotic resistant bacteria and why?  How likely is it that antibiotic treatment will lead to antibiotic resistant bacteria?  How do antibiotic resistant bacteria get into pet food?  What kind of pet food could be at risk for carrying antibiotic resistant bacteria?  Which food products could be an antibiotic resistant bacteria risk and why?  How likely is it that antibiotic resistant bacteria are transmitted from foods to humans?  How likely is it that antibiotic resistant bacteria through foods will lead to serious consequences?  In which countries/regions is the antibiotic resistant bacteria risk high and why?  How likely is it to get antibiotic resistant bacteria abroad?  How likely is it to become seriously ill from antibiotic resistant bacteria abroad?  Which of these transmission possibilities might be most relevant for you? When are negative consequences most likely? | |
| Preventive measures  Behavioural barriers | What is the problem about antibiotic resistant bacteria and the spread of antibiotic resistant bacteria?  What can be done as a protection against antibiotic resistant bacteria?  What can you do to protect your pet from antibiotic resistant bacteria? (e.g. food, antibiotic consumption, contact with animals/people)  *If not mentioned by the interviewee:* What can you do to prevent an antibiotic resistant bacteria transmission between you and your pet?  *If not mentioned by the interviewee:* How can you prevent the spread of antibiotic resistant bacteria through food at home?  *If not mentioned by the interviewee:* How can you prevent the transmission of antibiotic resistant bacteria abroad?  What reasons can you think of that could prevent people from implementing such behaviours when dealing with pets?  What barriers could you think of for people preparing food?  What barriers could you think of when people are abroad? | | What are the consequences of antibiotic resistant bacteria for people/animals?  Why is it helpful?  Which measures did you already implement and why?  Which measures aren`t you adopting and why?  What effect does (*mentioned behaviour*) have?  How difficult is it to implement (*mentioned behaviour*)?  Why is it difficult?  How effective are these measures?  What could be motivating?  What could be supportive? | |
| Intervention to reduce antibiotic resistant bacteria | Do you intent to do anything against the spread of antibiotic resistant bacteria in the future?  What do you need to implement such measures?  An online intervention (e.g. website) is developed with the aim of promoting the safe handling of pets and food in order to reduce the transmission of resistant bacteria. How should this intervention be designed?  **Interviewees had to make commentaries on ideas (e.g. facts or case study) on how to implement the intervention.**  What do you think of reminders of certain measures that can be easily integrated into everyday life? e.g. via the smartphone  Have you recently seen a campaign on food consumption and bacteria?  Now the last question: Where did you hear about antibiotic resistant bacteria? | | *If yes:* How will you implement this intention?  *If no:* Why not?  Which information could be useful?  Do you think consumers would us it?  Why would you use it?  Can you think of any other possibilities that could motivate people to change their behaviour?  What did you think about it?  Did you search for further information?  *If yes:* Why and where?  *If no:* Why not? | |

1. Interviewee can ask questions
2. Explain the study goal
3. Thank and hand over the compensation.

**S6.** Preventive measures mentioned by the food preparers to prevent AMR transmissions in the kitchen. Measures are listed in decreasing order according to the frequency they were mentioned. Corresponding recommendations by the Federal Food Safety and Veterinary Office (i.e. clean, separate, cook and chill) are indicated in brackets.

| **Implementation^#^** | **Preventive measures** |
| --- | --- |
| ✓☺ | Wash fruits/vegetables before preparing or eating them [clean] |
| ✓☺ | Chill required foods (mainly animal food products) [chill] |
| ✓ | Wash hands before eating/preparing food [clean] |
| ✓ | Heat the meat thoroughly [cook] |
| ✓ | Use a separate cutting board for chicken/meat [separate] |
| ✓ | Purchase organic or Swiss products |
|  | Keep an eye on the expiration date of food products |
|  | Clean the kitchen surface regularly [clean] |
| 🖐 | Cover or chill leftover food [chill] |
| 🖐 | Wash hands before and after preparing chicken [clean] |
|  | Clean or change dishcloths regularly [clean] |
|  | Wash all products before putting it in the fridge [clean] |
| 🖐 | Wash raw chicken [clean] |
|  | Strengthen your immune system so that AMR do not get a chance to cause disease |

^#^ Symbols represent the following types of implementation: ✓: Quoted by the majority (≥4) of food preparers. ☺: Consciously implemented by the majority (n≥4) of food preparers. 🖐: Implemented only by the participant who mentioned it (n=1).

**S7.** Preventive measures mentioned by pet owners to prevent AMR transmissions through their pets. Measures are listed in decreasing order according to the frequency they were mentioned.

| **Implementation^#^** | **Preventive measures** |
| --- | --- |
| ✓☺ | Strengthen pets` immune system |
| ✓☺ | Vaccinate the pet |
| ✓ | Do not bring your pet to bed |
|  | Avoid close contact to the pet |
|  | Do not feed raw meet |
| 🖐 | Do not allow the pet to lick your face |
| 🖐 | Train the dog not to eat everything |
| 🖐 | Wash hands after walking the dog |
|  | Reduce the pets` contact with other animals |
| 🖐 | Wash and groom the dog after going for a walk |
|  | Clean the cage regularly and wash pet blankets at 90 degrees Celsius |
|  | Use antiseptics |
|  | Use a separate water/food bowl for a sick animal |

^#^ Symbols represent the following types of implementation: ✓: Quoted by the majority (≥4) of pet owners. ☺: Consciously implemented by the majority (≥4) of pet owners. 🖐: Implemented only by the pet owner who mentioned it (=1).
